# Meeting report, “First Indian national conference on cervical cancer management - expert recommendations and identification of barriers to implementation”

**DOI:** 10.1186/s40661-018-0061-5

**Published:** 2018-07-26

**Authors:** K. S. Tewari, A. Agarwal, A. Pathak, A. Ramesh, B. Parikh, M. Singhal, G. Saini, P. V. Sushma, N. Huilgol, S. Gundeti, S. Gupta, S. Nangia, S. Rawat, S. Alurkar, V. Goswami, B. Swarup, B. Ugile, S. Jain, A. Kukreja

**Affiliations:** 1 Division of Gynecologic Oncology, Department of Obstetrics & Gynecology, University of California, Irvine, The City Tower, 333 City Blvd, West - Suite 1400, Orange, CA USA; 2 Medical Oncology, Dr. B.L. Kapur Memorial Hospital, New Delhi, India; 3 Cancer Care Hospital, Nagpur, Maharashtra India; 4 Apollo Speciality Hospital, Chennai, Tamil Nadu India; 5 Bombay Hospital, Mumbai, Maharashtra India; 6 Indraprastha Apollo Hospitals, New Delhi, India; 7 Max Super Speciality Hospital, Vaishali, India; 8 KIMS Hospital, Hyderabad, Telangana India; 9 Nanavati Hospital, Mumbai, Maharashtra India; 10 Nizams Institute Medical Sciences, Hyderabad, Telangana India; 11 Medanta Hospital, Gurgaon, Haryana India; 12 Indraprastha Apollo Hospital, Delhi, India; 13 Dharamshila Hospital, New Delhi, India; 14 Apollo Hospitals, Ahmedabad, Gujarat India; 15 Fortis Hospital, Noida, Uttar Pradesh India; 16 Roche Products (India) Pvt. Ltd., Mumbai, Maharastra India

**Keywords:** Cervical cancer, Cancer management, India, Expert opinion

## Abstract

**Objective:**

In India, cervical cancer accounts for almost 14% of all female cancer cases. Although poverty continues to cast a wide net over the Indian subcontinent, the preceding three decades have borne witness to improvements in nutrition and sanitation for many citizens. However, due to an absence of a national immunization program to cover human papillomavirus (HPV) vaccination and lack of accessible cervical cancer screening, the disease is characterized by late detection, lack of access to affordable and quality health care, and high mortality rates. Treatment of cervical cancer is stage-specific and depends on the patient’s age, desire to preserve fertility, overall health, the clinician’s expertise, and accessibility to resources. There is a paucity of uniform treatment protocols for various stages of cervical cancer in India. Considering all these parameters, a need to optimize treatment paradigms for the Indian population emerged.

**Methods/materials:**

Three expert panel meetings were held in different regions of India from 2016 to 2017. They were comprised of 15 experts from across the country, and included surgical oncologists, radiation oncologists, and medical oncologists. The panel members reviewed the literature from both national and global sources, discussed their clinical experience and local practices and evaluated current therapeutic options and management gaps for women diagnosed with cervical cancer.

**Results:**

This article summarizes the expert opinion from these meetings. It discusses the available resources and highlights the current therapeutic options available for different cervical cancer stages: early stage disease, locally advanced tumors, recurrent/persistent/metastatic cancer. An Indian consensus governing treatment options emerged, including guidelines for use of the only approved targeted therapy in this disease, the anti-angiogenesis drug, bevacizumab.

**Conclusions:**

The panel concluded that given the availability of state-of-the-art imaging modalities, surgical devices, radiotherapeutics, and novel agents in several population-dense urban centers, a uniform, multi-disciplinary treatment approach across patient care centers is ideal but not realistic due to cost and a paucity of third party payors for most Indian citizens. Preventative strategies including visual inspection with acetic acid to screen for precursor lesions (i.e., cervical intraepithelial neoplasia) with immediate referral for cervical cryotherapy and possible large-scale roll-out of the HPV vaccine in the near future can be expected to reduce mortality rates significantly in this country.

## Introduction

**Fig. 1:**
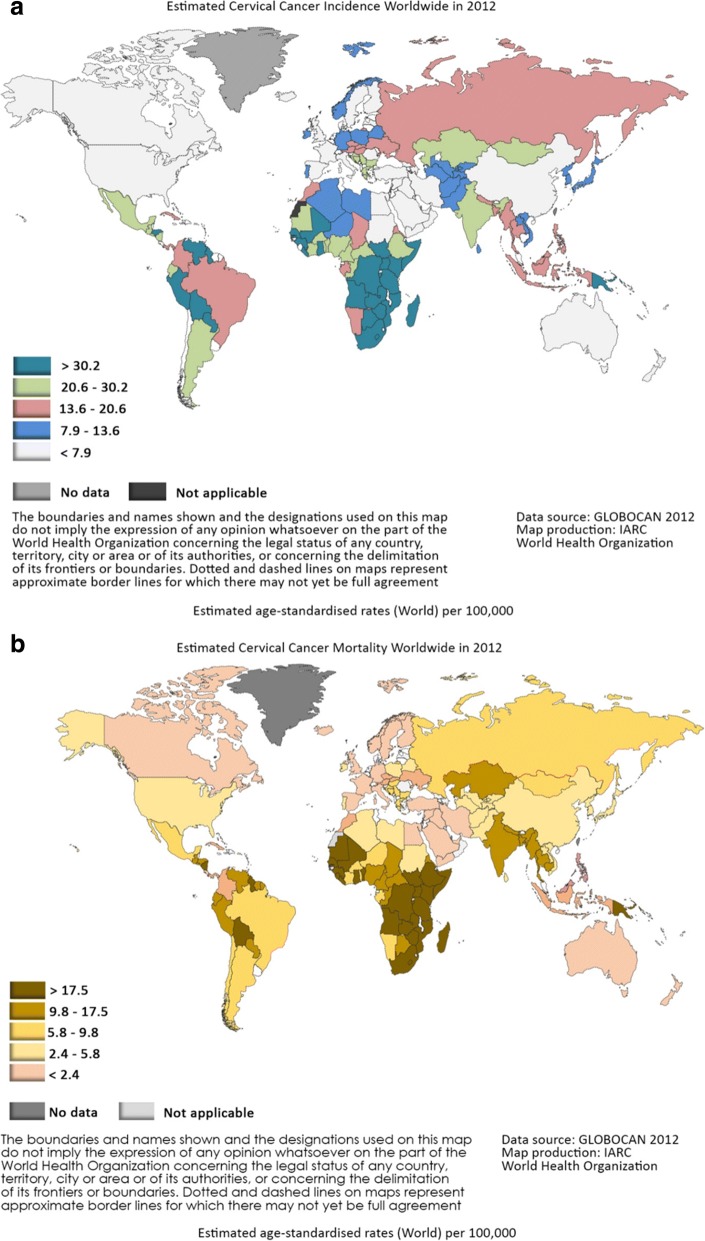
**a** depicts the estimated Cervical Cancer Incidence worldwide in 2012. **b** depicts the estimated Cervical Cancer Mortality worldwide in 2012

As per GLOBOCAN, cervical cancer is the fourth most common cancer in women with an estimated 528,000 new cases (Fig. 1a) and 266,000 deaths in 2012 (Fig. 1b) [1].

**Fig. 2:**
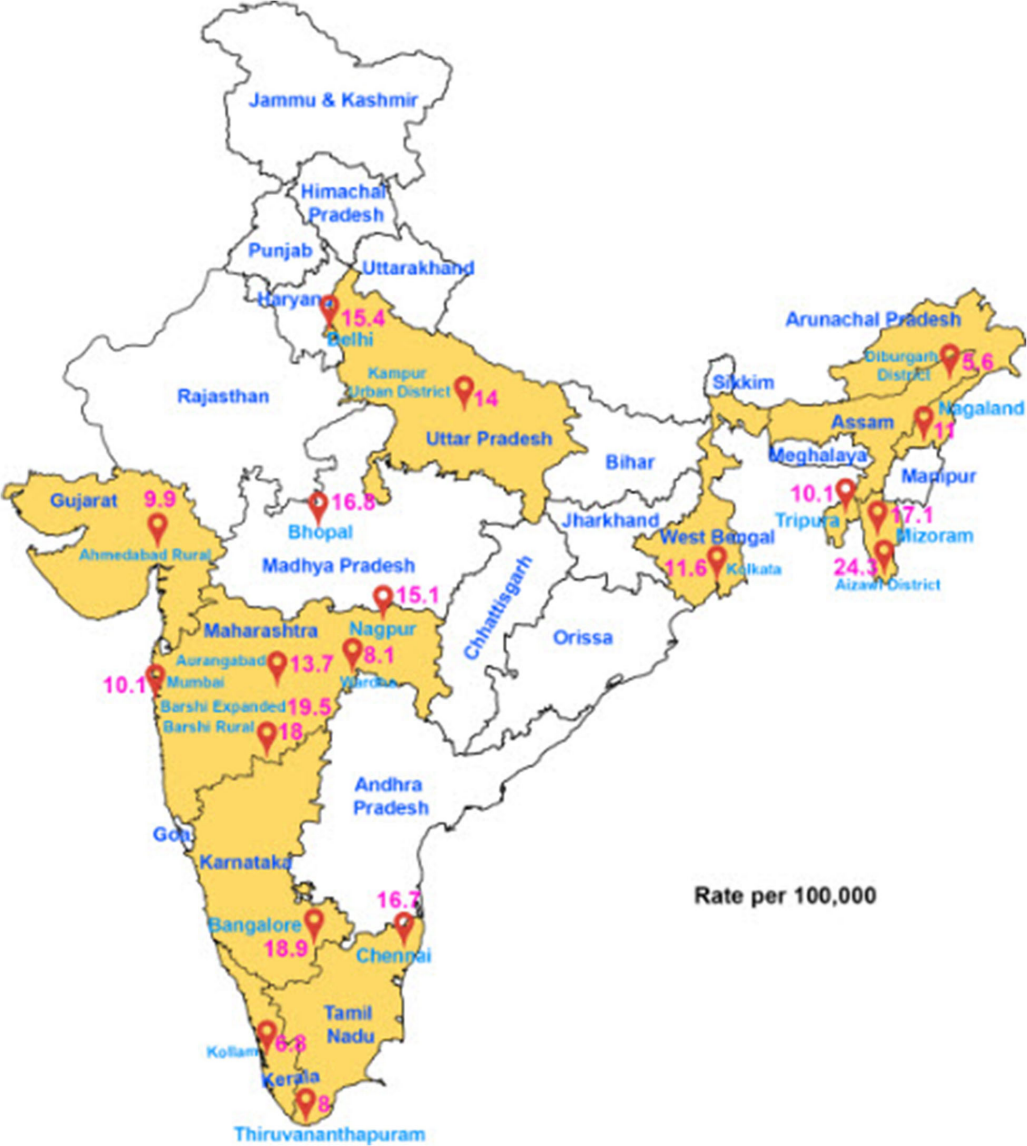
Age adjusted Incidence of Cervical Cancer in India (rate per 100,000) as per the Different Population Based Cancer Registries are depicted in a political map of India

In India, cervical cancer is the second most common cancer in women (aged 15–44 years) after breast cancer accounting for almost 14% of all female cancer cases [2, 3]. The age-adjusted incidence rate (AAIR) is 27.0 per 100,000 female population (Fig. 2) and age-adjusted mortality rate (AAMR) per 10,000 population is reported to be 12.4 [4, 5]. The higher mortality rate can be attributed largely to the lack of appropriate healthcare infrastructure in India [5, 6]. Cervical cancer in its advanced stage has a dismal outcome in terms of both prognosis and quality of life, registering approximately 67,477 deaths (23.3% of all cancer-related deaths) each year in Indian women [3, 7].

### Screening and immunization programs availability in India

Several screening (visual inspection with acetic acid [VIA], magnified VIA [VIAM], visual inspection with Lugol’s iodine [VILI], human papilloma virus [HPV] DNA testing and the Papanicolaou test) and diagnostic tests (cystoscopy, proctoscopy, examination under anesthesia) and imaging (computed tomography; CT; magnetic resonance imaging; MRI; positron emission tomography; PET scan; chest x-ray, and intravenous urography) are available for cervical cancer. However, their availability, specifically to patients residing in rural areas is limited [8–11].

Sankaranarayanan et al. (2009), in a cluster-randomized trial, assigned 131,746 healthy women aged 30 to 59 years to four groups (HPV testing, cytologic testing, visual inspection of the cervix with acetic acid [VIA], or standard care [control]). After 8 years of follow-up, the incidence rates of stage II or higher cervical cancer and death rates from cervical cancer were lowest in the HPV testing group. The hazard ratio (HR) for the detection of advanced cancer was 0.47 (95% confidence interval [CI]: 0.32–0.69) and for death was 0.52 (95% CI: 0.33–0.83) in the HPV-testing group when compared to the control group. In the other two experimental groups, significant reductions in the numbers of advanced cancers or deaths were not observed [12]. Further, Shastri et al., examined the feasibility and efficacy of VIA in reducing cervical cancer mortality by conducting a cluster-randomized study that included 151,538 women aged 35 to 64 years. After 12 years of follow-up, the VIA screening group showed 31% reduction in cervical cancer mortality when compared to the control group (rate ratios [RR]: 0.69; 95% CI: 0.54–0.88; *p* = 0.003) [13].

For cervical cancer, three types of vaccinations are already approved by the United States Food and Drug Administration which immunizes against various HPV types. They are HPV 2 (protects against subtype 16 and 18), HPV4 (6, 11, 16 and 18); and HPV9 (6, 11, 16, 18, 31, 33, 45, 52, and 58) [14]. With the exception of HPV immunization program in only two districts (Bathinda and Mansa) in the state of Punjab, currently there are no national HPV/cervical cancer immunization programs in India. The program has been launched with technical support from the World Health Organization (WHO) Country Office for India. First phase of the program has vaccinated approximately 10,000 girls of sixth standard in government schools of the above mentioned two districts, and is planned to be expanded to the other parts of the state with time [15].

### Treatment options for cervical cancer

The International Federation of Gynecology and Obstetrics (FIGO) stratifies cervical cancer in four stages and the treatment depends on the cancer stage. Certain other factors can also impact the treatment decision such as location and type of cancer (squamous cell cancer or adenocarcinoma), age, overall health, and the patient’s desire to have children [16]. Generally, early cancers are treated surgically, locally advanced cancers are treated with chemoradiation, and recurrent/metastatic cancers of the cervix may be salvaged with pelvic exenteration or palliated with systemic chemotherapy plus bevacizumab [16]. Apart from these, palliative care can be offered to patients to improve their quality of life and that of their respective families [17]. In India, cervical cancer is characterized by high incidence, late detection, lack of access to affordable and quality health care, and high mortality rates. Although, 360 million (30%) Indian population has taken up health insurance policies in the year 2015–2016, but there still remains a need to improve the awareness about health insurance policies among the rural communities where majority of the India’s population resides [18, 19]. This expert opinion aims to highlight the treatment paradigm of cervical cancer from an Indian perspective and aims to help in the effective management of these patients.

## Materials and methods

Three expert panel meetings were held at different regions in India. First two expert meetings were held at regional level in Delhi (Aug 6) and Hyderabad (June 18) in 2016, followed by a national level meeting in Mumbai in January 2017. Fifteen (15) members comprising of radiation oncologists, medical oncologists, surgical oncologists, and gynecologic oncologists were involved in this process (panel members listed in the Appendix). Although the meetings were supported by Roche, there were no Roche employees included among the expert panels physician roster. The expert opinion report was developed based on:

- Discussion by panel members who were convened to review the current therapeutic options and management gaps in cervical cancer patients
- A targeted review of literature from both national and global sources

This article highlights stage-specific cervical cancer treatment options, the Indian consensus and resource accessibility for the same.

## Results

### Treatment options, rationale for management, and recommendations specific to India

#### Early stage (FIGO stage IA-IB1 < 2 cm) desires fertility

For preservation of fertility, the National Comprehensive Cancer Network (NCCN) and the American Society of Clinical Oncology (ASCO) guidelines recommends cone biopsy with negative margins for stage IA cervical cancer without lymphovascular space invasion (LVSI). Whereas, for stage IA cancer with LVSI and stage IA2, cone biopsy with negative margins with pelvic lymph node dissection (PLND) or radical trachelectomy with PLND is recommended. Radical trachelectomy with PLND is also recommended for stage IB1 [20, 21]. As per ASCO guidelines, women’s with stage IB1 desiring fertility may also require adjuvant therapy if tumor is > 2 cm [21].

Patient survival with conization for stage IA cancer is similar to that with hysterectomy [22]. A Romanian study showed that radical vaginal trachelectomy and laparoscopic pelvic lymphadenectomy presents a safe therapeutic option in early stage cervical cancer with negligible recurrence rate and thus, promises to be a suitable option for young patients who want to retain their fertility [23]. Further, a Swedish study demonstrated that robotics-assisted laparoscopic radical trachelectomy in early stage cervical cancer patients is associated with high fertility rates (81%), low premature deliveries (6%), and an acceptable rate of tumor recurrence (4%) [24]. Studies have also shown that fertility sparing surgical management in the form of radical trachelectomy for early cervical cancer have a low rate of recurrence, few complications, and encouraging rates of conception and uneventful pregnancies although fertility treatment may be required [25, 26].

##### Indian consensus

The panelists unanimously agreed that early stage cervical cancer can be effectively managed by cone biopsy and radical trachelectomy. Cone biopsy with removal of pelvic lymph nodes and radical trachelectomy with PLND are the best treatment options for fertility preservation. Pelvic lymphadenectomy for fertility preservation can be successfully carried out with laparoscopy.

#### Early stage IA2 to IB1

When preservation of fertility is not desired, the NCCN and ASCO guidelines recommends performing extrafascial or modified radical hysterectomy with PLND or pelvic external beam radiation therapy (EBRT) plus brachytherapy [20].

##### Indian consensus

The panelists agreed that radical hysterectomy with PLND and tailored adjuvant radiotherapy/chemoradiation and/or brachytherapy should be recommended for women who do not desire fertility.

#### Early stage IA2 and IB1 (fertility not desired)

Guidelines recommend modified radical hysterectomy with PLND or pelvic EBRT plus brachytherapy for early stages IA2 and IB1, if fertility is not desired [20]. If surgery is chosen, it can be performed as an open procedure or using minimally invasive techniques (eg. laparoscopy, robotic-assisted laparoscopy). In addition, patients with IB1 stage are also suggested to be treated with adjuvant radiotherapy or concomitant chemo-radiotherapy (CCRT), if required [21]. Presence of intermediate risk factors (vascular and lymphatic permeation, tumor size > 2 cm, and deep cervical stroma invasion) or high-risk factors (positive pelvic lymph nodes, parametrial infiltration, and positive surgical margins) in surgically treated early-stage cervical cancer patients can dictate the use of adjuvant radiation or chemoradiation [27]. Adjuvant pelvic radiotherapy in intermediate-risk stage IB cervical cancer patients who underwent radical hysterectomy and pelvic lymphadenectomy showed 15% recurrence rate compared to 28% in the patient group who had no further treatment (*p* = 0.008) [28]. Similarly, high risk cervical cancer patients who received CCRT and pelvic radiation therapy after radical surgery showed improved progression free survival (PFS) and overall survival (OS) when compared to patients treated with adjuvant pelvic radiation therapy and surgery (PFS: 80% vs. 63%; *p* = 0.003 and Overall Survival (OS): 81% vs. 71%; *p* = 0.007) [29]. Adjuvant treatment with pelvic EBRT is indicated in case of large tumor size, more than one-third stromal invasion and/or LVSI (Sedlis Criteria) [20, 28]. It has been suggested that intensity-modulated radiation therapy (IMRT) may reduce the radiation dose to bowel and other vital structures by virtue of its ability to intensify dose to cancerous tissues while sparing the surrounding healthy tissue. IMRT can be used in patients post hysterectomy and also in the treatment of para-aortic nodes [30].

##### Indian consensus

Experts recommended adjuvant radiotherapy and cisplatin-based CCRT for cervical cancer patients with intermediate and high-risk factors for tumor recurrence. In cases where surgical expertise is unavailable, or the patient is unsuitable for surgery, radiation therapy with either intracavitary brachytherapy alone or along with external beam radiation therapy remains a viable treatment of choice.

#### Locally advanced disease IB2 to IVA

Treatment of locally advanced disease consists of pelvic EBRT with concurrent cisplatin-based chemotherapy and brachytherapy [20]. Table 1 present clinical studies assessing the efficacy and safety of chemotherapy and radiotherapy.

**Table 1:** Clinical Trials Assessing Efficacy and Safety of Chemotherapy and Radiotherapy in Locally advanced disease IB2 to IVA

Trials	Patient population	Treatment Arms	Results	Conclusions/Discussions
Morris et al. 1999 [31]	Cervical cancer confined to the pelvis (stages IIB through IVA or stage IB or IIA with a tumor diameter of at least 5 cm or involvement of pelvic lymph nodes)	Radiotherapy + chemotherapy (fluorouracil + cisplatin) *vs.* radiotherapy alone in high risk cervical cancer	5-year survival rate: 73% *vs*. 58%; *p* = 0.004	Addition of chemotherapy with fluorouracil and cisplatin to radiotherapy significantly improves survival rate
Kang et al. 2015 [32]	Patients will all tumor stages	Temporal treatment patterns for cervical cancer per guideline recommendations	Factors affecting likelihood of treatment per guidelines: age (*p* < 0.0001); tumor stage at diagnosis (*p* = 0.002) Reduction in all‑cause mortality: 56%; cancer related mortality: 49%	Treatment per guideline recommendations reduced mortality rates and OS
Au-Yeung et al. 2013 [33]	Patients with Locally advanced cervix cancer patients	Carboplatin + radiation for locally advanced cervical cancer	No significant benefit in OS or DFS	Concurrent use of carboplatin along with radiation therapy does not impact survival rate
Sebastiao et al. 2016 [34]	Patients with cervical cancer stage IIB-IVA	Cisplatin + radiotherapy *vs*. carboplatin + radiotherapy in advanced cervical cancer	3-year PFS: 59% *vs*. 40% 3-year OS: 70% *vs*. 68%	Overall, both cisplatin and carboplatin were similar with respect to 3-years OS, PFS, ORR, and toxic effects
Hashemi et al. 2013 [35]	Patients with cervical cancer stage IIB to stage IVA	Gemcitabine + cisplatin + radiotherapy	ORR: 97.3% 3-year DFS: 67% OS: 72%	Inconclusive regarding the benefit with gemcitabine. Further phase III studies required to validate the results
Narayan et al. 2016 [36]	Patients with stage I(IB2) and locally advanced (stages II-IVA) cervical cancer	NACT followed by CCRT *vs.* CCRT	DFS: 58.3% *vs*. 41.8%; *p*=0.001	Combination NACT with paclitaxel and cisplatin may improve long-term survival of patients with cervical cancer
Gill et al. 2015 [37]	Patients with stage IB1 to IVA cervical cancer	MRI-guided high-dose-rate intracavitary brachytherapy for cervical cancer	2-year local control: 91.6%; DFS: 81.8%; cancer-specific survival rate: 87.6%	Excellent local control and considerable morbidity was observed
Li et al. 2014 [38]	Patients with stage IIb-IVa cervical squamous cell carcinoma, adenocarcinoma, or adenosquamous carcinoma; seven patients had lymph node metastasis	Raltitrexed/cisplatin with concurrent radiotherapy; additional radiation was administrated to the lymph node metastases	OS: 90.7%	Favorable efficacy and acceptable adverse events

^Legend: *OS* Overall survival, *PFS* Progression free survival, *ORR* Overall response rate, *DFS* Disease free survival, *NACT* Neoadjuvant chemotherapy, *CCRT* Concurrent chemoradiation therapy, *MRI* Magnetic resonance imaging^

Lymphatic metastases are known to be higher in patients with locally advanced cervical cancer than in those at early stage. In spite of improved local control and OS with CCRT, almost 10–15% of the patients develop para-aortic lymph node (PALN) metastasis. Extended field irradiation combined with CCRT has shown better results in such cases. (Table 1) [38].

Extended-field CCRT is also an effective and a reasonable option for stage IIB-IVA cervical cancer patients with positive pelvic lymph nodes and radiologic negative PALN [39]. Internal radiation therapy/ intracavitary radiotherapy/ brachytherapy when combined with EBRT demonstrates good tolerance and is safe with acceptable morbidity [40, 41]. Brachytherapy is preferred after EBRT for radical treatment which delivers huge proportional radiation dose to the residual tumor while sparing the adjacent local organs (bladder and rectum) [42]. As per the International Commission on Radiation Units and Measurements (ICRU) report 38, brachytherapy can be administered at low-, medium-, and high-dose rate [43]. Higher dose is mandatory to achieve local control and poor tumor geometric conditions may require interstitial brachytherapy [44, 45].

Brachytherapy can be high dose rate (HDR) or low dose rate (LDR). In LDR brachytherapy, cesium-137 isotope is used, and a point A dose rate of < 0.4 Gray/hour is delivered. HDR brachytherapy uses iridium-192 isotope and a point A dose rate of > 12 Gray/hour. Although HDR is gaining more popularity in the recent years, the overall results and toxicity with HDR and LDR are considered to be comparable [46].

Main indications for interstitial brachytherapy include large tumors, lower vaginal involvement, lateral extension of disease, and ill-fitting intracavitary applicators [46]. Younger women can be offered the option for laparoscopic ovarian transposition to move ovaries out of radiation field. This helps in reducing the exposure to radiations by 90% [47].

##### Indian consensus

The panel members jointly agreed that locally advanced cancers are best treated with CCRT. Adjuvant chemotherapy may be considered as per the clinician’s perspective. Chemotherapy is for the systemic management while radiotherapy limits local disease. The choice of therapeutic regimen is as follows: cisplatin+ paclitaxel > > carboplatin + paclitaxel > cisplatin alone > > cisplatin + gemcitabine. The experts had a detailed discussion regarding the sequence of the two management options: radiotherapy and chemotherapy and finally it was concluded that the sequence of administering the modalities depends on the patient’s condition, availability of radiotherapeutic units and the clinician’s perspective. It was also agreed that the choice of treatment for a PALN negative stage IIB-IVA cervical cancer patient would include CCRT to the pelvis plus brachytherapy, and for a PALN positive patient, CCRT to pelvis with extended field radiotherapy plus brachytherapy.

#### Metastatic (stage IVB) disease

The prognosis of metastatic cervical cancer is usually poor and the main objectives of treatment include slowing the cancer growth and relieving the symptoms. The management of stage IVB will be addressed together with the management of persistent and recurrent disease later in the paper.

##### Indian consensus

The panel members unanimously agreed that systemic chemotherapy is mandatory for advanced stage cervical cancer patients while radiotherapy is essential for improvement of their symptoms including vaginal bleeding, pelvic pain, pain due to bone metastases etc. Cisplatin, carboplatin (chemotherapeutic agents), and bevacizumab (targeted therapy) are the available treatment choices. Considering increased toxicity, topotecan is not recommended for the management of advanced stage cervical cancer and the preferred therapeutic choice is cisplatin + paclitaxel + bevacizumab.

#### Persistent disease

The management of persistent disease along with metastatic and recurrent disease will be addressed later.

##### Indian consensus

Adding bevacizumab to chemotherapy for persistent cervical cancer improves OS, progression free survival (PFS) and overall response rate (ORR). Combination therapy with cisplatin, paclitaxel and bevacizumab is superior than topotecan, paclitaxel, and bevacizumab regimen similar to that for metastatic (stage IV B) disease.

#### Recurrent disease-isolated central recurrence

Pelvic recurrence of cervical cancer is categorized into three categories; they are central pelvic, lateral pelvic and extra-pelvic. The management of central pelvic recurrence varies with the previous treatment received by the patient i.e. radical hysterectomy without adjuvant irradiation or only irradiation. Women’s treated with irradiation in the past are only left with pelvic exenteration as a surgical therapeutic option [48]. NCCN also states that cancers recurring centrally (in the pelvis only) might see a better response with pelvic exenteration while in patients with recurrence at distant locations (such as lungs or bone), radiation and chemotherapy may be used [20].

A Korean study showed that with pelvic exterentation, the 5-year OS and 5-year DFS were 56 and 49% for metastatic cases [49]. Similarly, an Indian study reported a good 5-year OS (> 55%) in patients who underwent pelvic exenteration [50]. However, both the studies reported high morbidity rates associated with pelvic exenteration (44 and 62.50%, respectively) [49, 50].

Further, it is recommended that these patients should then undergo a pelvic reconstructive procedure which include bowel reconstruction, urinary reconstruction, and vagina reconstruction [51]. The reconstructive procedures are reported to lower morbidity rates. Continent diversions are preferred as they have been found to improve quality of life [52]. However, they too are associated with complications such as ureteral stricture/obstruction, difficult catheterization and pyelonephritis [53]. These patients require to be given post-operative care in intensive care units (ICUs). A patient is estimated to spend two to three days on an average in Intensive Care Unit post-surgery where regular care by the team involving intensive care team, colorectal surgeons for primary care, surgical specialty review, stoma therapy, nursing, and other health staff is required [54].

##### Indian consensus

The panelists stated that pelvic exenteration may provide the opportunity of long-term survival in carefully selected cervical cancer patients. They also highlighted that pelvic exenteration is a morbid procedure creating a huge financial and psychological burden.

#### Recurrent disease non-exenteration candidate, first-line therapy

Although cisplatin administered intravenously (50 mg/m^2^) every 3 weeks is considered the most effective single agent in the treatment of metastatic cervical cancer, patients who have already received cisplatin as a radio sensitizer, may not respond to the single drug regimen. Therefore, cisplatin-based combination therapies are used in these patients [55–57]. According to Pfaendler and Tewari (2016), the standard treatment for recurrent cancer patients is cisplatin plus paclitaxel [58].

Now-a-days studies are being conducted to assess the effectiveness of adding bevacizumab (vascular endothelial growth factor-specific angiogenesis) inhibitor to chemotherapeutic regimen in patients with recurrent, persistent, or metastatic cervical cancer. Recurrence after prior chemotherapy results in very poor prognosis of cervical cancer patients can be better dealt by incorporating bevacizumab in the treatment regime.

NCCN guidelines gives class 1 recommendation for bevacizumab to be given as first line treatment (in following combinations: cisplatin/paclitaxel/bevacizumab; topotecan/paclitaxel/bevacizumab; carboplatin/paclitaxel/bevacizumab) in patients with metastatic or recurrent cervical cancer [20].

Patients with metastatic disease may experience symptoms of vaginal bleeding, pelvic pain, pain due to bone metastases etc. Palliative radiotherapy must be used based on the site of metastases, patient’s performance status and life expectancy, and potential treatment toxicity. Evidence suggests that longer duration of radiotherapy may not be required as short courses are as effective [59, 60].

#### Indian consensus

Experts agreed that without evidence of inferiority, carboplatin and paclitaxel are an alternative for patients with recurrent disease, unless they have not received prior chemoradiation therapy. In patients with renal dysfunction, carboplatin is better tolerated owing to its milder toxic profile. Adding bevacizumab to chemotherapy for recurrent cervical cancer improves OS, PFS and ORR as in patients with persistent disease. The combination therapy with cisplatin, paclitaxel and bevacizumab appears to be clinically more feasible than topotecan, paclitaxel and bevacizumab regimen.

#### Recurrent disease, second-line therapy

Kamura et al. (2013) in his review concluded that chemotherapy can be considered as a second line treatment for patients with recurrent cervical cancer, but is less effective due to drug resistance. A phase II trial has assessed the efficacy and tolerability of bevacizumab in patients with recurrent or persistent cervical cancer. The study results showed median PFS and OS times to be 3.40 months (95% CI: 2.53–4.53 months) and 7.29 months (95% CI: 6.11–10.41 months), respectively. Further, phase III clinical trials are warranted. It has suggested bevacizumab to be used as second or third line drug in patients with recurrent cervical cancer [61]. NCCN guidelines also recommend bevacizumab for second line treatment of recurrent cervical cancer in addition to other drugs (category 2B unless otherwise indicated) including docetaxel, 5-FU, gemcitabine, ifosfamide, mitomycin, irinotecan, albumin bound paclitaxel (i.e., nab-paclitaxel), topotecan, pemetrexed, and vinorelbine [20]. Chemotherapy is the only choice for a palliative therapy in patients with recurrent non-central cervical cancer who had previously been treated with surgery plus adjuvant irradiation and chemo (radiation) [48]. NCCN guidelines states that patients who had recurrence even after second line treatment (surgery or radiation therapy) can be given “chemotherapy or best supportive care, or can be enrolled in a clinical trial.” [20]. It should be noted that in June 2018, the anti-PD-1 checkpoint inhibitor, pembrolizumab, was granted accelerated approved in the United States by the U.S. Food and Drug Administration for second-line therapy in women with recurrent cervical cancer. It is too early to determine the likelihood of availabilty of this drug and other immuno-oncology agents for this indication in India.

##### Indian consensus

The panel agreed that palliative chemotherapy should be given to patients with recurrent cervical cancer as second line treatment. The need of best supportive care for these patients was also suggested.

#### Resources and feasibility

Availability of resources for management of cervical cancer in India is presented in Table 2.

**Table 2:** Availability of Resources for Management of Cervical Cancer in India

Resources	Availability in India
Loop Electrocautery Excision Procedure	It is a safer procedure as compared to cold knife conization, but is not very common in India as it requires expensive machines [62].
Radical trachelectomy	It is an expensive surgical option, thus unaffordable by women in poor-resource setting who are known to be more commonly diagnosed with cervical cancer.
Surgical expertise	As per the ASCO resource-stratified clinical practice guideline, fertility sparing treatment requires surgical expertise which may not be easily available in the basic or limited settings [63].
Radiotherapy	There is a one radiotherapy machine per 2-5 million cancer patients which highlights the lack of accessibility to radiotherapy. The use of brachytherapy in India is limited due to following reasons. • Lack of radiation oncologists and radiotherapy technologists. • Private hospitals favour other techniques including IMRT and IGRT over brachytherapy. • Implementation of latest advancements in brachytherapy is limited to a few premier hospitals [64]. However, despite all these challenges, the use of brachytherapy has shown a surge in India.
Chemotherapy	Chemotherapeutic drugs are available in India, but the associated cost is high. • A recent observational study by Kolasani *et al.* (2016) assessed the variation in prices of anti-cancer drugs (chemotherapy) in India. Physicians might not prescribe the low-priced drug due to lack of information on quality, conflict of interest, and a belief that new drug is better than the older [65]. • Thus, if awareness is raised among the treating physicians about the quality of the low-priced drugs, it will increase the accessibility and affordability of the treatment to lower and middle-class patients in India. • Pharmaceutical companies and government should also make efforts to reduce the drug prices to lower the economic burden on the patients. [64]
CCRT	Annually, 38,771 patients with cervical cancers in India do not receive CCRT, resulting in poorer survival. [66]
HDR and interstitial brachytherapy	• Ir-192 is commonly used for HDR brachytherapy in state government funded hospital in India, but its replacement with Co-60 will be a cost-effective option in developing countries like India. [64] • Few studies in Indian settings have assessed the applicability of interstitial brachytherapy in patients not suitable to undergo intracavitary brachytherapy. [67–69]
Bevacizumab	• The cost-effectiveness analysis of bevacizumab has been conducted using the Markov decision tree in 240 patients enrolled in the GOG trial. The study concluded that ICER associated with bevacizumab could be reduced by introducing biosimilars, and/or other cheaper and efficacious anti-angiogenesis agents. [70] • Roche is currently running 'The Blue Tree' program for cancer patients in India. This program covers several aspects to help cancer patients ranging from “diagnostics, funding of treatment, information, post-treatment job search, assistance with documentation for reimbursement and free medicines where possible.”
Pelvic exenteration procedure	• This procedure requires tremendous economic and psychosocial support which a developing country like India largely lacks. • Poverty and illiteracy further makes optimal rehabilitation of these patients difficult [50]. • There is a need of increase in beds at ICUs in both private and public hospitals with the expected rise in number of cancer patients. The associated high cost of ICUs in private hospitals and lack of its coverage under health insurance, the oncology patients has to borrow or sell assets for admission to ICUs [71].
Palliative Care	• There is a huge lack of manpower in Indian hospitals to provide palliative care to cancer patients [72]. • Thus, it is not possible to provide palliative care to all cancer patients in India.

Abbreviations: *ASCO* American Society of Clinical Oncology, *IMRT* Intensity-modulated radiotherapy, *IGRT* Image-guided radiotherapy, *Ir* Iridium, *CCRT* Concurrent radiotherapy, *Co* Cobalt, *ICER* Incremental cost-effectiveness ratio, *ICU* Intensive care units

## Discussion

Treatment of cervical cancer is stage-specific and depends on patient’s age, desire to preserve fertility, overall health, clinician’s expertise, and accessibility to resources. This expert opinion highlights the current therapeutic options available for different cancer stages (early-, advanced, and recurrent) and Indian consensus on their treatment options.

Majority of the early-stage cancers are treated with surgery which successfully preserves the fertility without compromising treatment and survival.

The stage-wise best management practices followed in India are presented in Table 3.

**Table 3:** Summary of Cervical Cancer Management Practices Followed in India

Cone biopsy and radical trachelectomy with pelvic lymph node dissection are the best treatment option for fertility preservation in early stage (IA1, IA2, IB1 and IB2) cervical cancer patients.	
Patients not interested to maintain fertility can undergo radical hysterectomy with PLND and tailored adjuvant radiotherapy/chemoradiation and/or brachytherapy.	
Stage IA2-IB1 cervical cancer patients are typically treated with radical hysterectomy with PLND with or without para-aortic lymph node sampling. They are further managed with adjuvant CCRT depending on the surgico-pathologic findings. Radiation therapy includes both EBRT and brachytherapy.	
In a developing country like India, radiotherapeutic facilities are limited and generally patients have a lengthy waiting period, hence, neo-adjuvant chemotherapy with cisplatin and paclitaxel is the preferred alternative for early stage cervical cancer patients. The high cost of chemotherapeutic agents increases the economic burden on the patients.	
For stage IB2-IVA, primary CCRT plus brachytherapy with or without adjuvant cisplatin or carboplatin based chemotherapy is an effective management option	
Cervical cancer stage IVB is incurable and the main treatment option is palliation. Incorporation of bevacizumab with chemotherapy doublets may improve survival by a median 3.7 months.	
Pelvic exenteration may be curative for a patient with a central, isolated recurrence but if the patient is not an exenteration candidate (eg., non-central recurrence or metastatic disease or refuses exenteration).	

Legend: *PLND* Pelvic lymph node dissection, *CCRT* Concurrent chemoradiation therapy, *EBRT* External beam radiation therapy

The targeted therapies, like bevacizumab, which have demonstrated clinical benefits to these patients (in clinical studies as well as clinical experience of some of the Indian experts) need to be widely utilized in India, as per this expert group’s opinion.

## Conclusion

The treatment of cervical cancer is stage-specific and necessitates cognizance of patient’s age, desire to preserve fertility, overall health, clinician’s expertise, and accessibility to healthcare infrastructure. The panel concluded that a uniform, multi-disciplinary treatment approach across patient care centers using advanced therapeutics should be widely utilized in India. The panel acknowledges that prevention represents the most feasible and effective path forward but that requires commitment to develop a VIA screening infrastructure and national endoresment of a HPV vaccination program.

## Abbreviations

AAIR: Age-Adjusted Incidence Rate
AAMR: Age-Adjusted Mortality Rate
ASCO: American Society of Clinical Oncology
CCRT: Concomitant Chemo-Radiation Therapy
CI: Confidence Interval
CT: Computed Tomography
EBRT: External Beam Radiation Therapy
FIGO: International Federation of Gynecology and Obstetrics
HDR: High Dose Rate
HPV: Human Papilloma Virus
HR: Hazard Ratio
ICRU: International Commission on Radiation Units and Measurements
IMRT: Intensity-Modulated Radiation Therapy
LDR: Low dose rate
LVSI: Lymphovascular Space Invasion
MRI: Magnetic Resonance Imaging
NCCN: National Comprehensive Cancer Network
ORR: Overall Response Rate
OS: Overall Survival
PALN: Para-Aortic Lymph Node
PET: Positron Emission Tomography
PLND: Pelvic Lymph Node Dissection
RR: Rate Ratios
VIA: Visual Inspection with Acetic Acid
VIAM: Visual Inspection with Acetic Acid Magnified
WHO: World Health Organization
